# A Multimodal Artificial Intelligence Model to Guide Use of Whole-Pelvic Radiation Therapy in Patients with Localized Prostate Cancer: Exploratory Analysis of RTOG 9413

**DOI:** 10.3390/cancers18121982

**Published:** 2026-06-18

**Authors:** Mutlay Sayan, Huei-Chung Huang, Erin L. Stewart, Timothy N. Showalter, Adam P. Dicker, George Daniel Grass, Elizabeth M. Gore, Andrew Michael McDonald, J. Daniel Pennington, Mark A. Hallman, Igor J. Barani, I-Chow Hsu, Michael Rooney, Stephanie L. Pugh, Paul L. Nguyen, Phuoc T. Tran, Mack Roach III

**Affiliations:** 1 Mass General Brigham and Dana-Farber Cancer Institute, Boston, MA 02115, USA; 2 Artera, Los Altos, CA 94022, USA; 3 UVA Comprehensive Cancer Center, Charlottesville, VA 22908, USA; 4 Thomas Jefferson University, Philadelphia, PA 19107, USA; 5 H. Lee Moffitt Cancer Center and Research Institute, Tampa, FL 33612, USA; 6 Clement J Zablock VAMC and the Medical College of Wisconsin, Milwaukee, WI 53295, USA; 7 University of Alabama at Birmingham, Birmingham, AL 35294, USA; 8 Southeast Clinical Oncology Research Consortium NCORP, Winston Salem, NC 27103, USA; 9 Virginia Urology, Richmond, VA 23235, USA; 10 Fox Chase Cancer Center, Philadelphia, PA 19111, USA; 11 St. Joseph’s Hospital & Medical Center, Phoenix, AZ 85013, USA; 12 The University of Texas MD Anderson Cancer Center, Houston, TX 77030, USA; 13 NRG Oncology Statistics and Data Management Center, Philadelphia, PA 19103, USA; 14 UCSF Medical Center-Mount Zion, San Francisco, CA 94115, USA

**Keywords:** artificial intelligence, prostatic neoplasms, radiotherapy

## Abstract

**Background/Objectives**: This study explored whether a multimodal artificial intelligence (MMAI) model integrating digitized histopathology and clinical features can identify prostate cancer patients who may benefit from neoadjuvant hormonal therapy (NHT) and whole-pelvic radiotherapy (WPRT). **Methods**: This secondary analysis of NRG/RTOG 9413 included NHT-treated patients with digitized biopsy slides and clinical data who were not part of the MMAI model optimization. A previously validated MMAI model estimated long-term risk, and Fine-Gray models evaluated interactions between MMAI-derived scores and the radiation field (WPRT vs. prostate-only RT [PORT]) for biochemical failure (BF), chosen over progression-free survival because of extended follow-up and distant metastasis (DM), with subgroup analyses by predefined MMAI strata. **Results**: Among 81 eligible patients, the MMAI-by-treatment interaction for BF did not confirm a differential effect (*p* = 0.30). Therefore, subgroup findings should be interpreted as descriptive and hypothesis-generating. Nevertheless, the magnitude effect of WPRT was numerically greater in the MMAI high-risk subgroup (5-yr: 41% vs. 79%; 10-yr: 47% vs. 79%; aHR 0.35 [0.14–0.86]) than in the low–intermediate group (5-yr: 18% vs. 33%; 10-yr: 44% vs. 57%; aHR 0.66 [0.29–1.48]). **Conclusions**: Although no statistically significant treatment-by-MMAI interaction was demonstrated, these findings are hypothesis-generating and support further investigation of MMAI approaches for guiding WPRT in NRG/RTOG 0534, 0924, GETUG-01, and POP-RT trials.

## Simple Summary

Whole-pelvic radiotherapy may improve outcomes for some patients with localized prostate cancer, but identifying who is most likely to benefit remains challenging. In this study, we evaluated whether a multimodal artificial intelligence model that combines information from biopsy tissue images and clinical characteristics could identify patients with different outcome patterns following whole-pelvic versus prostate-only radiotherapy. Using data from a randomized clinical trial, we found that patients classified as high risk by the model showed larger numerical differences in biochemical failure rates with whole-pelvic radiotherapy, although the formal statistical interaction test was not significant. These findings should be considered exploratory and hypothesis-generating. Nevertheless, these findings support further investigation of artificial intelligence-based risk assessment as a tool for risk stratification and hypothesis generation in future prospective clinical trials.

## 1. Introduction

External-beam radiotherapy (EBRT) with hormonal therapy (HT) is a standard of care for intermediate- and high-risk prostate cancer [1,2,3,4,5,6,7,8]. Still, the role of whole-pelvic RT (WPRT) remains debated, especially regarding which patients are likely to benefit. RTOG 9413 demonstrated a progression-free survival (PFS) benefit with WPRT versus prostate-only RT (PORT) [9], with long-term follow-up confirming durable benefit among patients receiving neoadjuvant-HT (NHT) [10]. However, GETUG-01 did not show a PFS improvement with WPRT [11,12]. Recent data highlight the importance of patient selection, including high-risk disease [13] and younger age [14]. Current risk stratification tools lack precision in identifying who benefits from WPRT [15,16]. Multimodal artificial intelligence (MMAI) algorithms may help by integrating clinical variables and digitized histopathology [17]. While these models have been validated in predicting clinical outcomes [18,19,20,21,22], their potential role in informing treatment selection—particularly for WPRT—remains unexplored.

In this study, leveraging patient-level data from RTOG 9413, we applied a previously validated MMAI prognostic model to explore whether MMAI-derived risk groups exhibit distinct patterns of biochemical or metastatic outcomes following NHT with WPRT versus PORT. This secondary analysis is intended to provide insights that may motivate validation in trials such as POP-RT, GETUG-01, NRG/RTOG 0534, and 0924.

## 2. Materials and Methods

### 2.1. Study Cohort

This retrospective analysis included consenting patients enrolled in the NRG/RTOG 9413 trial, which compared WPRT versus PORT and NHT versus adjuvant-HT in localized prostate cancer. Details of the trial design have been previously described [9,10,23,24].

The current analytic cohort excluded patients used in the MMAI model development (v1.2) [25]. This cohort corresponds to the internal validation group used in MMAI model development and was further reduced to patients receiving NHT; the subgroup guided the design of NRG/RTOG 0924 [26,27].

### 2.2. Data Acquisition

Digitized H&E-stained biopsy slides and clinical data were obtained for a subset of NRG/RTOG 9413 patients with pretreatment biopsy images, complete clinical data, and no prior use in model optimization. Slides were scanned at 20× magnification and underwent centralized quality assurance.

### 2.3. Application of the MMAI Model

We applied a validated MMAI prognostic model integrating biopsy histopathology with clinical data (PSA, age, and clinical T-stage) to generate a prognostic risk score [17,20,25]. This model was trained and validated across multiple randomized trials and demonstrated superior prediction performance for distant metastasis (DM) and biochemical failure (BF) compared to conventional tools. The model combined convolutional neural network-extracted image features with clinical variables through a fully connected neural network. The model combined image-derived features from biopsy histopathology with clinical variables using a neural network.

### 2.4. Statistical Analysis

The endpoints of interest were BF and DM, analyzed using time-to-event methods. BF was selected over the trial’s original PFS endpoint due to the extended duration of follow-up, during which PFS events increasingly reflected non-cancer-related deaths [10], limiting its utility in assessing prostate cancer-specific outcomes. BF was chosen for its clearer signal of disease control and treatment effect, while DM was included because it is the endpoint toward which the MMAI model was initially developed.

To explore whether BF or DM differed by MMAI between WPRT and PORT, Fine-Gray models with interaction terms between treatment and MMAI were used. Correspondingly, estimated treatment effects were adjusted for biomarker interaction (adjusted hazard ratio, aHR), and 95% confidence intervals (CIs) were reported. Subgroup summaries were generated within MMAI risk strata (low–intermediate vs. high) using previously validated thresholds (low risk: <0.273; intermediate risk: 0.273–0.499; and high risk: >0.499). Cumulative incidence functions accounting for competing risks (defined as death without an event) were used to provide BF and DM risk estimates for the MMAI subgroups and comparisons between treatment groups (WPRT vs. PORT) based on Gray’s test were treated as descriptive and not intended for inference.

All tests were two-sided at a 0.05 significance level, without multiplicity adjustments. Given the exploratory design, findings should be considered hypothesis-generating.

## 3. Results

**Figure 1:**
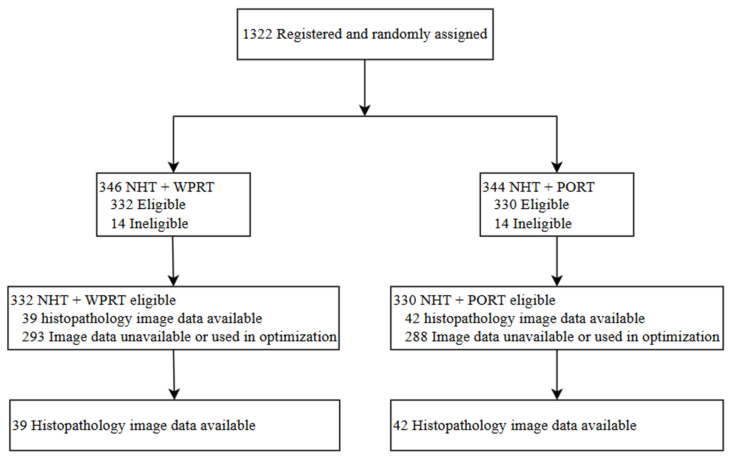
CONSORT diagram. Abbreviations: NHT, neoadjuvant hormonal therapy; PORT, prostate only radiotherapy; WPRT, whole-pelvic radiotherapy.

**Figure 2:**
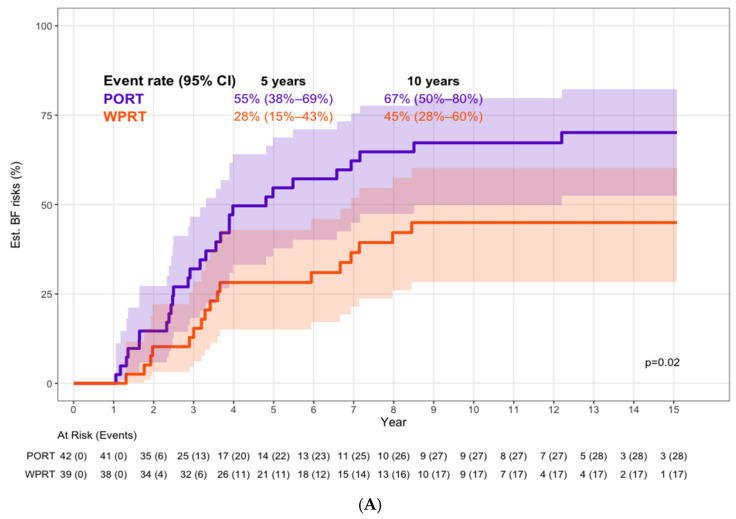

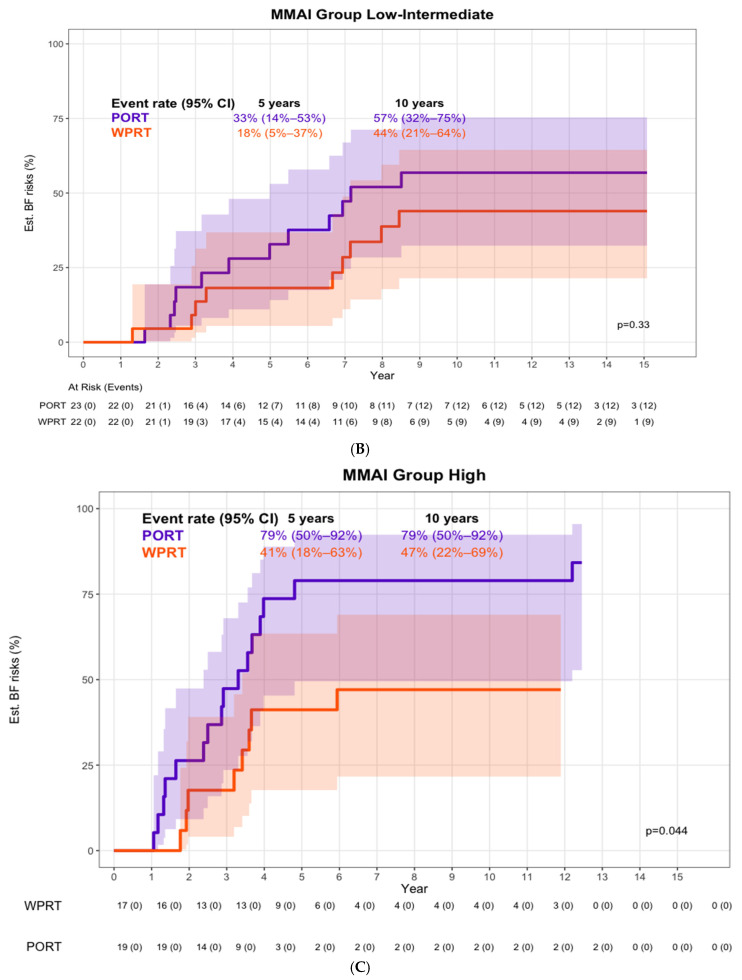

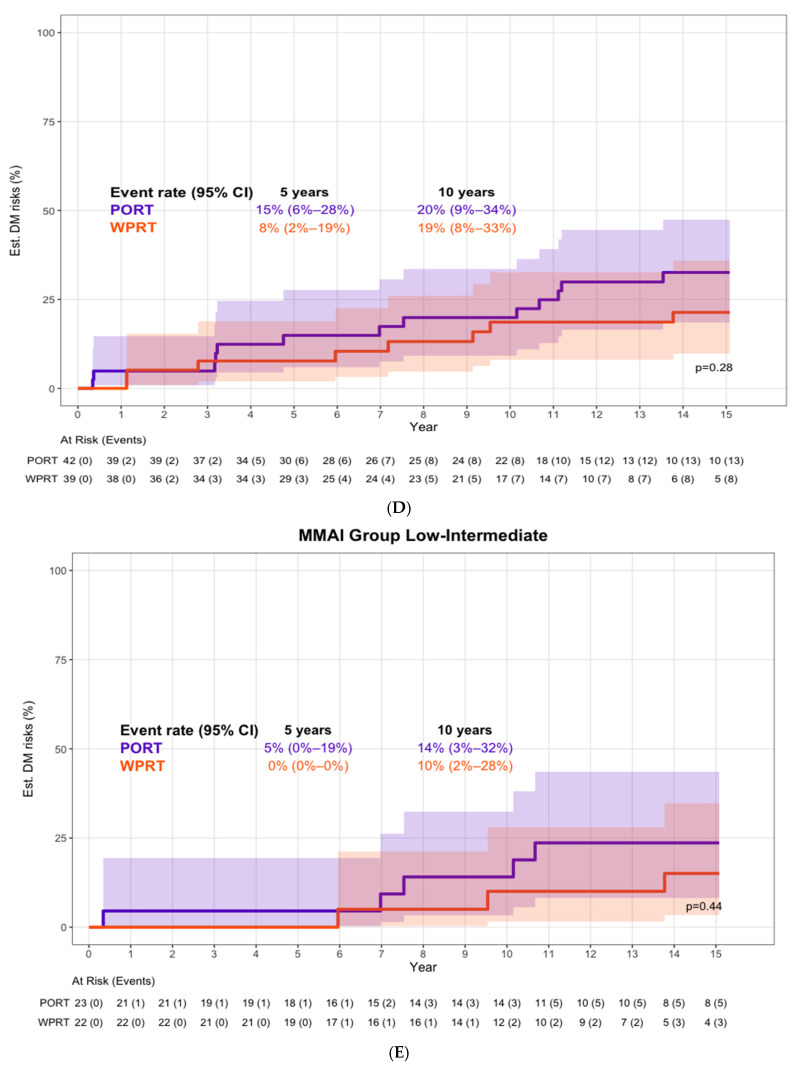

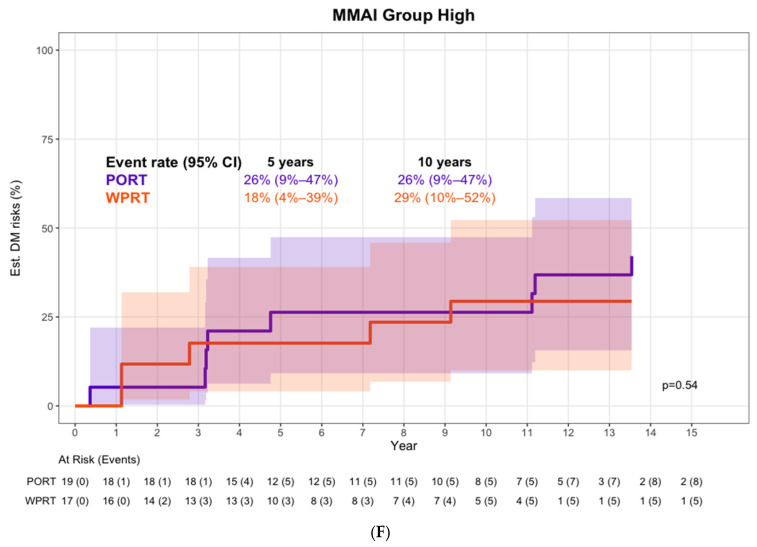
Cumulative incidence estimates and 95% confidence bands of biochemical failure (**A**–**C**) and distant metastasis (**D**–**F**) among patients who received neoadjuvant hormonal therapy by MMAI risk classification and radiation treatment volume. Shaded areas represent the 95% confidence bands around each cumulative incidence curve.

Eighty-one patients were included in this analysis (Figure 1; Table 1). BF rates were lower with WPRT (5-year: 28% vs. 55%; 10-year: 45% vs. 67%; aHR 0.49 [0.27–0.89]). The treatment-by-MMAI interaction was not statistically significant (interaction *p* = 0.30), indicating no evidence of treatment effect heterogeneity in this cohort. Accordingly, all subgroup findings should be interpreted as descriptive and hypothesis-generating. Nevertheless, exploratory subgroup estimates showed numerically larger risk differences between WPRT and PORT among patients classified as MMAI high-risk (5-year: 41% vs. 79%; 10-year: 47% vs. 79%; exploratory-aHR 0.35 [0.14–0.86]) than among those in the low–intermediate group (5-year: 18% vs. 33%; 10-year: 44% vs. 57%; exploratory-aHR 0.66 [0.29–1.48]) (Figure 2). For DM, no apparent differences were observed between WPRT and PORT (5-year: 8% vs. 15%; 10-year: 19% vs. 20%; exploratory-aHR 0.62 [0.26–1.48]). The interaction between the MMAI category and the radiation field on DM was assessed (interaction *p* = 0.82). WPRT vs. PORT treatment effects were aHR 0.56 [0.14–2.25] for MMAI low–intermediate subgroup vs. aHR 0.69 [0.22–2.16] for MMAI high subgroup (Figure 2). Because baseline PSA differed numerically between treatment groups, a sensitivity analysis was performed by adding PSA as a continuous covariate to the interaction models. For BF, the treatment-by-MMAI interaction estimate remained similar and was modestly strengthened after PSA adjustment (primary model: sHR 0.52, *p* = 0.30; PSA-adjusted model: sHR 0.45, *p* = 0.20). For DM, results were essentially unchanged (primary model: sHR 1.22, *p* = 0.82; PSA-adjusted model: sHR 1.20, *p* = 0.85). Exploratory NCCN risk group analyses are presented in Supplementary Table S1. These analyses yielded unstable estimates because only 13 patients were classified as NCCN intermediate-risk and events were sparse.

## 4. Discussion

This exploratory analysis of NRG/RTOG 9413 examined whether MMAI-derived risk groups showed differing treatment effect patterns for WPRT versus PORT. Numerically larger BF differences were observed among MMAI high-risk patients than in the low–intermediate group, whereas DM estimates were similar across strata. However, the treatment-by-MMAI interaction was not statistically significant; therefore, these findings should be interpreted as descriptive and hypothesis-generating. Further retrospective evaluation in larger randomized datasets will be needed to determine whether these exploratory patterns are reproducible.

Despite validation of prior nodal risk models [28], more sensitive, biologically driven methods are needed to guide WPRT. The clinical relevance of these findings lies in the potential of MMAI risk classification to meet this unmet need, offering an efficient tool to personalize treatment decisions and inform future validation efforts.

These findings arrive at a time when the optimal use of WPRT remains uncertain, with randomized trials yielding inconsistent results. RTOG 9413 demonstrated a PFS benefit with WPRT [9,10], while GETUG-01 did not [11,12], and POP-RT showed BF benefits but lacks long-term data [13]. Recent observational work also suggests benefit in biologically selected subgroups [14]. Our analysis extends these insights by applying a validated MMAI framework.

Importantly, these findings should be considered within the context of contemporary PSMA-PET staging. Although PSMA-PET has substantially improved detection of nodal and distant metastases compared with conventional imaging, its sensitivity for microscopic pelvic nodal disease remains imperfect. Surgical validation studies have demonstrated that a meaningful proportion of patients with negative PSMA-PET findings harbor occult pathologic nodal metastases [29,30,31]. In this setting, tissue-based MMAI approaches may provide information complementary to imaging by capturing biologic features associated with aggressive disease and patterns of disease progression that are not constrained by imaging resolution. Rather than replacing PSMA-PET, MMAI may ultimately provide complementary information, with PSMA-PET identifying radiographically detectable disease and MMAI providing additional biologic risk stratification. Future studies should evaluate whether integrating MMAI with PSMA-PET improves risk stratification and further refines the study of WPRT in randomized clinical trial datasets.

NRG/RTOG 0534 and 0924 trials provide opportunities to assess whether MMAI can refine patient selection for WPRT and serve as a biomarker to guide treatment intensification. Additional validation opportunities may emerge from ongoing pelvic nodal radiotherapy trials, including PIVOTALboost and PEARLS. Future efforts should include prospective validation in larger randomized datasets adequately powered to test interactions. Using the observed interaction effect as a planning assumption, approximately 500 patients (corresponding to roughly 275 BF events) may be required to achieve 80% power for treatment-by-MMAI interaction testing.

Several limitations warrant consideration. This was a retrospective analysis, and treatment was not stratified by MMAI risk. Only a small subset (*n* = 81) of the original NHT cohort was available because many patients were used for original model optimization or lacked biopsy slides. Additionally, staging was based on pre-PSMA PET imaging, which may limit its applicability to modern practice. Moreover, while BF is a clinically relevant endpoint, its long-term association with prostate cancer-specific and overall mortality remains variable, particularly in the contemporary era of salvage therapies [32,33]. Nonetheless, BF occurred frequently enough in this cohort to permit more stable descriptive estimation of outcome patterns. In contrast, events for DM, PCSM, or OS were too sparse to support reliable subgroup summaries. Similarly, exploratory interaction analyses using NCCN risk groups yielded unstable estimates because only 13 patients were classified as NCCN intermediate-risk and events were sparse, resulting in clinically implausible treatment effect estimates that should be interpreted with caution (Supplementary Table S1). Given the limited sample size, all treatment effect estimates, particularly within MMAI risk strata, should be interpreted cautiously, and no confirmatory conclusions regarding treatment effect heterogeneity can be drawn.

BF was selected over the trial’s original primary endpoint of PFS because the extended follow-up of RTOG 9413—now exceeding two decades—led many progression events to be driven by non-cancer-related deaths rather than PSA progression, and neither the original trial nor the subset analyzed here was powered to assess DM or OS. As a result, PFS has become increasingly difficult to interpret as a cancer-specific measure of treatment efficacy. BF, by contrast, offers a more direct signal of disease control and treatment effect, consistent with its use in recent trials such as POP-RT and NRG/RTOG 0924.

## 5. Conclusions

This study observed a stronger association between WPRT and reduced BF among MMAI high-risk patients than among low- and intermediate-risk patients. Because no significant treatment-by-MMAI interaction was observed, these findings should be considered exploratory and hypothesis-generating. MMAI may offer a potential avenue for exploring personalized treatment selection beyond conventional risk stratification. These results motivate further adequately powered validation to support the broader integration of digital pathology-based AI tools in future clinical trial design.

## Figures and Tables

**Table 1:** Baseline characteristics of patients who received neoadjuvant hormonal therapy, stratified by radiation treatment volume.

Characteristic	Overall (*N* = 81)	PORT (*N* = 42)	WPRT (*N* = 39)	*p*-Value
Age, years, median (IQR)	70 (66–74)	70 (66–74)	70 (65–74)	
Race, No. (%)				0.6
African American	23 (28%)	13 (31%)	10 (26%)	
White	4 (4.9%)	1 (2.4%)	3 (7.7%)	
Other	54 (67%)	28 (67%)	26 (67%)	
PSA at baseline, median (IQR)	25.3 (11.8–38.3)	27.0 (16.8–40.2)	15.4 (9.8–33.7)	
Clinical Gleason Score, No. (%)				0.7
≤6	20 (25%)	9 (21%)	11 (28%)	
7 (3 + 4)	27 (33%)	14 (33%)	13 (33%)	
7 (4 + 3)	14 (17%)	9 (21%)	5 (13%)	
8	12 (15%)	5 (12%)	7 (18%)	
9–10	8 (9.9%)	5 (12%)	3 (7.7%)	
Clinical T Stage, No. (%)				0.4
T1c–T2a	17 (21%)	11 (26%)	6 (15%)	
T2b–c	36 (44%)	20 (48%)	16 (41%)	
T3/T3a	13 (16%)	5 (12%)	8 (21%)	
T3b–T4	15 (19%)	6 (14%)	9 (23%)	
Clinical N stage, No. (%)				0.6
N0	4 (4.9%)	3 (7.1%)	1 (2.6%)	
Nx	77 (95%)	39 (93%)	38 (97%)	
NCCN risk group, No. (%)				0.7
Intermediate	13 (16%)	6 (14%)	7 (18%)	
High	68 (84%)	36 (86%)	32 (82%)	
Follow-up, years, median (IQR)	14.5 (6.0, 17.0)	14.8 (8.5–18.3)	14.5 (4.9–17.0)	0.6

Abbreviations: PORT, prostate-only radiation therapy; WPRT, whole-pelvic radiation therapy; PSA, prostate specific antigen; NCCN, National Comprehensive Cancer Network.

## Data Availability

All data will be made available in accordance with the National Clinical Trials Network Data Archive policies and procedures.

## Supplementary Materials

The following supporting information can be downloaded at https://www.mdpi.com/article/10.3390/cancers18121982/s1: Table S1: Exploratory NCCN risk group analyses, neoadjuvant ADT cohort (n = 81). (A) NCCN by radiation field interaction effect; (B) Subgroup-specific treatment effects (WPRT vs. PORT) within each NCCN risk group to aid clinical interpretation of the interaction effect.

## Abbreviations

The following abbreviations are used in this manuscript:

ADT	Androgen deprivation therapy
aHR	Adjusted hazard ratio
BF	Biochemical failure
CI	Confidence interval
DM	Distant metastasis
EBRT	External-beam radiotherapy
H&E	Hematoxylin and eosin
HT	Hormonal therapy
IQR	Interquartile range
MMAI	Multimodal artificial intelligence
NCCN	National Comprehensive Cancer Network
NHT	Neoadjuvant hormonal therapy
OS	Overall survival
PCSM	Prostate cancer-specific mortality
PFS	Progression-free survival
POP-RT	Prostate-Only Versus Whole-Pelvic Radiation Therapy trial
PORT	Prostate-only radiotherapy
PSA	Prostate-specific antigen
PSMA PET	Prostate-specific membrane antigen positron emission tomography
RT	Radiotherapy
WPRT	Whole-pelvic radiotherapy
