# Supplementary material for: A Multimodal Artificial Intelligence Model to Guide Use of Whole-Pelvic Radiation Therapy in Patients with Localized Prostate Cancer: Exploratory Analysis of RTOG 9413
Source: Cancers (Basel). 2026 Jun 18;18(12):1982. doi: 10.3390/cancers18121982 (PMC13296454; doi:10.3390/cancers18121982)
Supplement: Supplementary file 1 [file cancers-18-01982-s001.zip › cancers-4328552-supplementary.pdf]

Supplementary Table S1: Exploratory NCCN risk group analyses, neoadjuvant ADT cohort (n=81). A) NCCN by radiation field interaction effect; B) Subgroup-specific treatment effects (WPRT vs. PORT) within each NCCN risk group to aid clinical interpretation of the interaction effect.

	**sHR (95% CI) (BF)**	**sHR (95% CI) (DM)**
**A. NCCN by radiation field interaction effect**		
NCCN High vs. Intermediate (ref)	3.68 (0.91–14.90)	Not estimable
WPRT vs. PORT (within NCCN Intermediate[1])	2.30 (0.42–12.44)	
NCCN High × WPRT (interaction)	0.16 (0.03–0.99)	
**B. Subgroup-specific treatment effects (WPRT vs. PORT) within each NCCN risk group**		
Intermediate[1] (n=13)	2.16 (0.45–10.42)	Not estimable
High (n=68)	0.37 (0.19–0.71)	0.47 (0.18–1.23)

Abbreviations: sHR, subdistribution hazard ratio; BF, biochemical failure; DM, distant metastasis; WPRT, whole-pelvic radiotherapy; PORT, prostate-only radiotherapy.
All estimates are highly unstable due to sparse data and should not be interpreted as informative. [1]NCCN Intermediate is the reference group (n=13); estimates within this subgroup are particularly unreliable. WPRT vs. PORT effect estimated within the NCCN Intermediate reference group. DM models did not produce stable estimates due to complete or nearly complete separation of events.

.
